# Clinical Significance of Anatomic Peculiarities and Ultrasound-Guided Electromyography of the Posterior Tibial Muscle

**DOI:** 10.7759/cureus.18719

**Published:** 2021-10-12

**Authors:** Fatma Elleuch, Wafa Elleuch, Harbi Houcem, Sameh Ghroubi, Habib M Elleuch

**Affiliations:** 1 Department of Physiology, Habib Bourguiba Hospital, University of Sfax, Sfax, TUN; 2 Deaprtment of Physical Medicine and Rehabilitation, Habib Bourguiba Hospital, University of Sfax, Sfax, TUN; 3 Department of General Surgery, Habib Bourguiba Hospital, University of Sfax, Sfax, TUN; 4 Department of Physical Medicine and Rehabilitation, Habib Bourguiba Hospital, University of Sfax, Sfax, TUN; 5 Department of Physical Medicine and Rehabilitation & Research Laboratory LR20ES09, Habib Bourguiba Hospital, University of Sfax, Sfax, TUN

**Keywords:** foot drop, posterior tibial muscle, ultrasound, electromyography, steppage gate

## Abstract

The posterior tibial muscle (PTM) is a key muscle in diagnosing the level of the neurologic lesion that causes steppage gate that is a paralysis of nervous origin of the muscles ensuring the foot dorsiflexion.

The aim of this manuscript is to illustrate the anatomical peculiarities of the PTM, the ultrasound (US) anatomy of the infero-posterior part of the leg, and the technique of US-guided electro-neuro-myography (ENMG) of the PTM, which is considered a key muscle in the diagnosis of the neurological lesion causing steppage gate.

The US-guided ENMG of the PTM is technically easy and safe for young practitioners provided there is a good knowledge of US anatomy of the infero-posterior part of the leg.

## Introduction

Nowadays, the needle electro-neuro-myography (ENMG) plays a major role in the site diagnosis of neurological lesions of the limbs, for example in steppage gate. The latter is also known as drop foot that is a paralysis of nervous origin of the muscles ensuring the dorsiflexion of the foot.

But this type of exploration requires a lot of expertise when it comes to deep nerve or muscle that has crucial contribution to diagnosis because of its anatomical peculiarities.

The use of ultrasound (US) facilitates the implementation of the ENMG of these anatomical elements and makes it accessible to less-experienced clinicians [1].

The aim of this manuscript is to illustrate the anatomical peculiarities of the posterior tibial muscle (PTM), the US anatomy of the infero-posterior part of the leg, and the technique of US-guided ENMG of the PTM, which is considered a key muscle in the diagnosis of the neurological lesion causing steppage gate.

## Technical report

A good knowledge of the US anatomy of the leg makes it easy to locate the PTM so as to prick it with ENMG needle.

**Figure 1:**
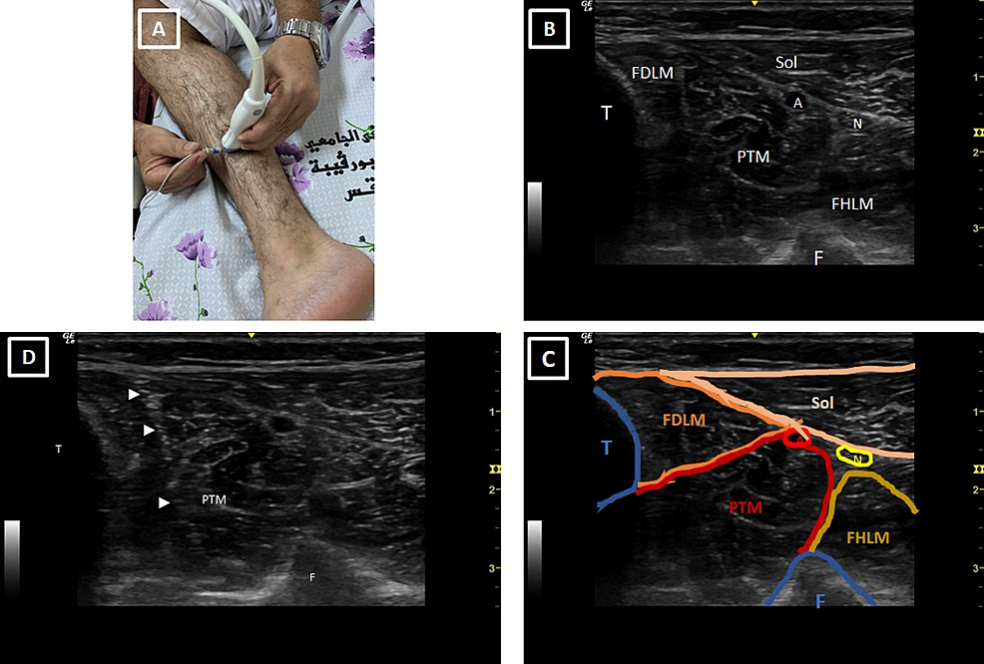
Technique of ultrasound-guided ENMG of the posterior tibial muscle (A) Placement of the ENMG needle under ultrasound control in the PTM. The ultrasound probe is placed in the middle of the posteromedial side of the leg. (B, C) Ultrasound sections in the middle part of the leg illustrating the different anatomical elements and locating the PTM. (D) Ultrasound view showing the ENMG needle (arrow heads) in the PTM. ENMG: electro-neuro-myography; T: tibia; F: fibula; PTM: posterior tibial muscle; Sol: soleus muscle; FDLM: flexor digitorum longus muscle; FHLM: flexor hallucis longus muscle; A: posterior tibial artery; N: posterior tibial nerve.

Indeed, a physician must first place the US probe in the middle third of the posteromedial side of the leg as shown in Figure 1A. The obtained US image should show the two bones of the leg centered by the PTM and part of the muscles of the anterior compartment of the leg (Figures 1B, 1C). It is then easy to prick the PTM by the ENMG needle under US guidance as shown in Figures 1A, 1D.

## Discussion

Anatomy reminder

The PTM is a deep muscle in the posterior compartment of the leg. It originates from the interosseous membrane and from the two bones of the leg: the proximal 2/3 of the medial face of the body of the fibula and of the dorsal face of the body of the tibia. Then it follows a slightly oblique path downwards and inwards up to the ankle where it is continued by a tendon which goes behind the medial malleolus in the gutter of the flexor muscles then passes above the sustentaculum tali, along the internal surface of the navicular bone to end at the plantar surface of the latter giving expansions toward all the bones of the foot except the talus, the first and the fifth metatarsals [2].

This muscle has several roles, namely plantar flexion, supination, and adduction of the foot. The combination of the last two movements results in the inversion of the foot. In addition, this muscle also helps maintain the arch or the hollow of the foot.

The PTM is innervated by the posterior tibialis nerve, which is a branch of the sciatic nerve. However, the PTM has the anatomical peculiarity of being the only muscle of the posterior compartment of the leg to be innervated by the L5 root like the foot levator muscles of the anterolateral compartment of the leg.

US contribution for performing ENMG

Several studies have proven the indisputable utility of US guidance for performing ENMG.

Precise ENMG needle placement is possible with US guidance for testing of nerves (particularly deep ones) and it is obviously superior to surface recordings of sensitive nerves [3-5].

Standard ENMG may show the absence of any motor unit potential (MUP) in patients with muscle plegia due to nerve trauma. But the US is very helpful for detection of isles of muscle contractility that should have MUP in ENMG and this has a clear prognostic impact [6].

US guidance has also proven to be useful in challenging ENMG of certain deep muscles as diaphragm muscle, teres minor muscle, and external anal sphincter [7-9].

Diagnostic value of the PTM

Steppage (also known as drop foot) is a paralysis of nervous origin of the muscles ensuring the dorsiflexion of the foot (also known as the elevator muscles of the foot and toes). These muscles are the tibialis anterior, extensor digitorum longus, extensor hallucis longus, and third fibularis. Any damage or compression of the nerve axis anywhere along its path has the potential to cause the foot to fall and steppage gate [10,11].

Involvement of the foot elevator motor nerve can be localized either at the level of the L5 root or at the level of the peroneal nerve (also known as the external popliteal sciatic nerve [EPS]). Consequently, in case of steppage gate it is mandatory to eliminate L5 root damage, which can perfectly simulate EPS damage without any radicular pain [12].

Taking into account that PTM is innervated by the L5 root but does not depend on the peroneal nerve, its clinical, US, and electromyographic evaluation is of great help in the diagnosis of the level of neurological lesion causing the steppage gate [12].

## Conclusions

Thanks to its anatomical peculiarities, the PTM is a key muscle in determining the level of the neurologic lesion that causes steppage gate. Its exploration by standard ENMG requires a lot of expertise but has been revolutionized by US, which has greatly facilitated and made it accessible to young practitioners provided they have a good knowledge of US anatomy of the infero-posterior part of the leg.
